# Supplementary material for: Synergistic activity of RSL3 and Pyrimethamine to inhibit the proliferation of Plasmodium falciparum
Source: Antimicrob Agents Chemother. 2025 Aug 18;69(10):e00471-25. doi: 10.1128/aac.00471-25 (PMC12486841; doi:10.1128/aac.00471-25)

LEGENDS TO SUPPLEMENTAL FIGURES

Supplemental Figure 1: Asynchronously growing *P. falciparum* parasites were treated with RSL3 and Dihydroartemisinin (DHA) at the indicated concentrations. After 72 hours, parasite growth was assessed by measurement of the total DNA content (SYBR Green staining). The values obtained from each biological replicate were plotted individually for each concentration of DHA. Corresponding to **Figure 1A**

Supplemental Figure 2: Treatment with RSL3 and Atovaquone as described in Suppl. Fig. 1. Corresponding to **Figure 1B**

Supplemental Figure 3: Treatment with RSL3 and Pyrimethamine as described in Suppl. Fig. 1. Corresponding to **Figure 1C**

Supplemental Figure 4: *P. falciparum* parasites were synchronized by sorbitol treatment. Late-stage parasites were treated with RSL3 and Pyrimethamine at the indicated concentrations for 48 hours. As a readout for parasite growth, the activity of *P. falciparum* LDH was measured. The values obtained from each biological replicate were plotted individually for each concentration of Pyrimethamine. Corresponding to **Figure 1E**

Supplemental Figure 5: Sustainable inhibition of *P. falciparum* growth by RSL3 and Pyrimethamine, second and third biological replicate. Corresponding to **Figure 2**

- **A.** *P. falciparum* parasites were treated with RSL3 as in Figure 2A (second replicate).
- **B.** Parasites were exposed to Pyrimethamine as in Figure 2B (second replicate).
- **C.** Treatment with RSL3 and Pyrimethamine as in Figure 2C (second replicate).
- **D.** *P. falciparum* parasites were treated with RSL3 as in Figure 2A (third replicate).
- **E.** Parasites were exposed to Pyrimethamine as in Figure 2B (third replicate).
- **F.** Treatment with RSL3 and Pyrimethamine as in Figure 2C (third replicate).

Supplemental Figure 6: Treatment with RSL3 and Cycloguanil hydrochloride as described in Suppl. Fig. 1. Corresponding to **Figure 4B**

Supplemental Figure 7: Treatment with RSL3 and Cycloguanil hydrochloride as described in Suppl. Fig. 4. Corresponding to **Figure 4D**

Supplemental Figure 8: Treatment with RSL3 and WR99210 as described in Suppl. Fig. 1. Corresponding to **Figure 5A**

Supplemental Figure 9: Treatment with RSL3 and Methotrexate as described in Suppl. Fig. 1. Corresponding to **Figure 5B**

Supplemental Figure 10: Impact of *1S,3R*-RSL3 and *1R,3R*-RSL3 on the intraerythrocytic proliferation of *P. falciparum*. Corresponding to **Figure 6B**

A. Parasite growth in response to the indicated concentrations of *1S,3R*-RSL3 was assessed by total DNA content measurement using the SYBR Green I fluorescence-based proliferation assay. The values obtained from each biological replicate were plotted.

B. *P. falciparum* parasites were treated with *1R,3R*-RSL3 and their proliferation was evaluated as described in (**A**).

C. Quantification of parasite growth (percentage) from three biological replicates for both *1S,3R*-RSL3 and *1R,3R*-RSL3.

Supplemental Figure 11: Treatment with *1R,3R*-RSL3 and Pyrimethamine as described in Suppl. Fig. 1. Corresponding to **Figure 6C**

Supplemental Figure 12: Treatment with Pyrimethamine and ML162 as described in Suppl. Fig. 1. Corresponding to **Figure 7A**

Supplemental Figure 13: Treatment with Pyrimethamine and Auranofin as described in Suppl. Fig. 1. Corresponding to **Figure 7B**

Supplemental Figure 14: Treatment with Pyrimethamine and TRi-1 as described in Suppl. Fig. 1. Corresponding to **Figure 7C**

Supplemental Figure 15: Treatment with Pyrimethamine and Hydroxyurea as described in Suppl. Fig. 1. Corresponding to **Figure 7D**

Supplemental Figure 16: Treatment with Pyrimethamine and Didox as described in Suppl. Fig. 1. Corresponding to **Figure 7E**

Supplemental Figure 17: Treatment with Pyrimethamine and Erastin as described in Suppl. Fig. 1. Corresponding to **Figure 8A**

Supplemental Figure 18: Treatment with Pyrimethamine and Erastin2 as described in Suppl. Fig. 1. Corresponding to **Figure 8B**

Supplemental Figure 19: Treatment with Pyrimethamine and *L*-Buthionine-*S,R*-sulfoximine (BSO) as described in Suppl. Fig. 1. Corresponding to **Figure 8C**

Supplemental Figure 20: Treatment with Pyrimethamine and Darapladib as described in Suppl. Fig. 1. Corresponding to **Figure 8D**

Supplemental Figure 21: Treatment with Pyrimethamine and Conoidin A as described in Suppl. Fig. 1. Corresponding to **Figure 8E**

Supplemental Figure 22: Lack of synergy between RSL3 and Pyrimethamine in the apicomplexan parasite *Toxoplasma gondii*

- **A.** Vero cells were infected with *T. gondii*, strain RH-LacZ. Cells were treated with RSL3 and Pyrimethamine at the indicated concentrations at 30 minutes post infection (p.i.). At 72 hours p.i., β-galactosidase activity was measured using chlorophenol red-β-D-galactopyranoside (CPRG) as a substrate. CPRG levels were measured by absorbance measurements at 570 nm, and represented as absorption units [AU]. Single-replicate values are displayed in Suppl. Fig. 23.
- **B.** Vero cells were infected with *T. gondii*, strain RH-pTUB-tdTomato RFP. Cells were treated with RSL3 and Pyrimethamine at the indicated concentrations at 30 minutes post infection (p.i.). Fluorescence as a measure of *T. gondii* RH-pTUB-tdTomato RFP

growth was determined at 138 hours p.i. The mean fluorescence intensity (MFI) is presented as units of $1x10^4$. Single-replicate values are displayed in Suppl. Fig. 24.

Supplemental Figure 23: No synergy between RSL3 and Pyrimethamine in *T. gondii*, strain RH-LacZ. Values obtained from single biological replicates were plotted individually for each concentration of Pyrimethamine. Corresponds to **Suppl. Fig. 22A**

Supplemental Figure 24: Lack of synergy between RSL3 and Pyrimethamine in *T. gondii*, strain RH-pTUB-tdTomato RFP. Values obtained from single biological replicates were plotted individually for each concentration of Pyrimethamine. Corresponds to **Suppl. Fig. 22B**

Supplemental Figure 1

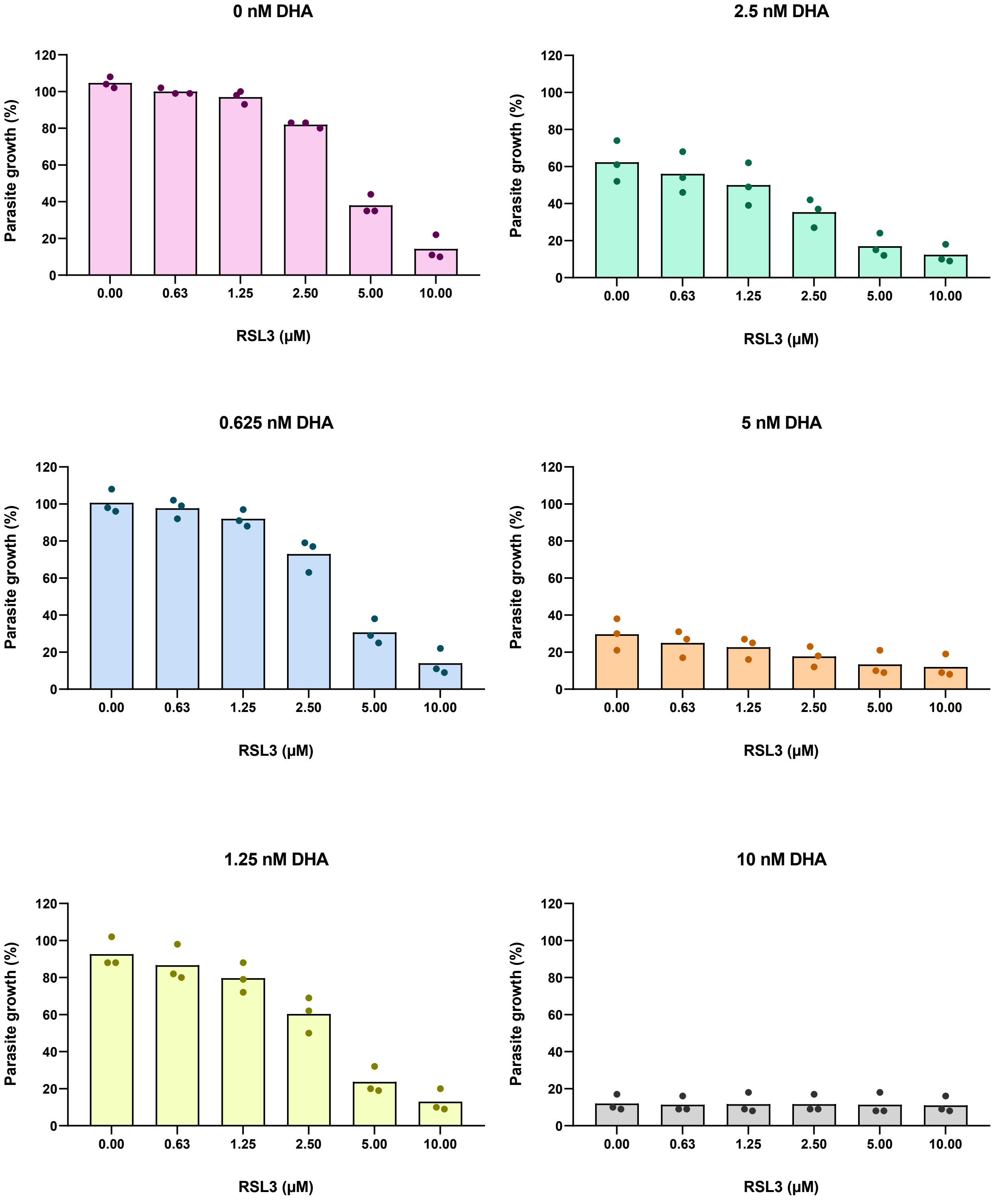

Supplemental Figure 2

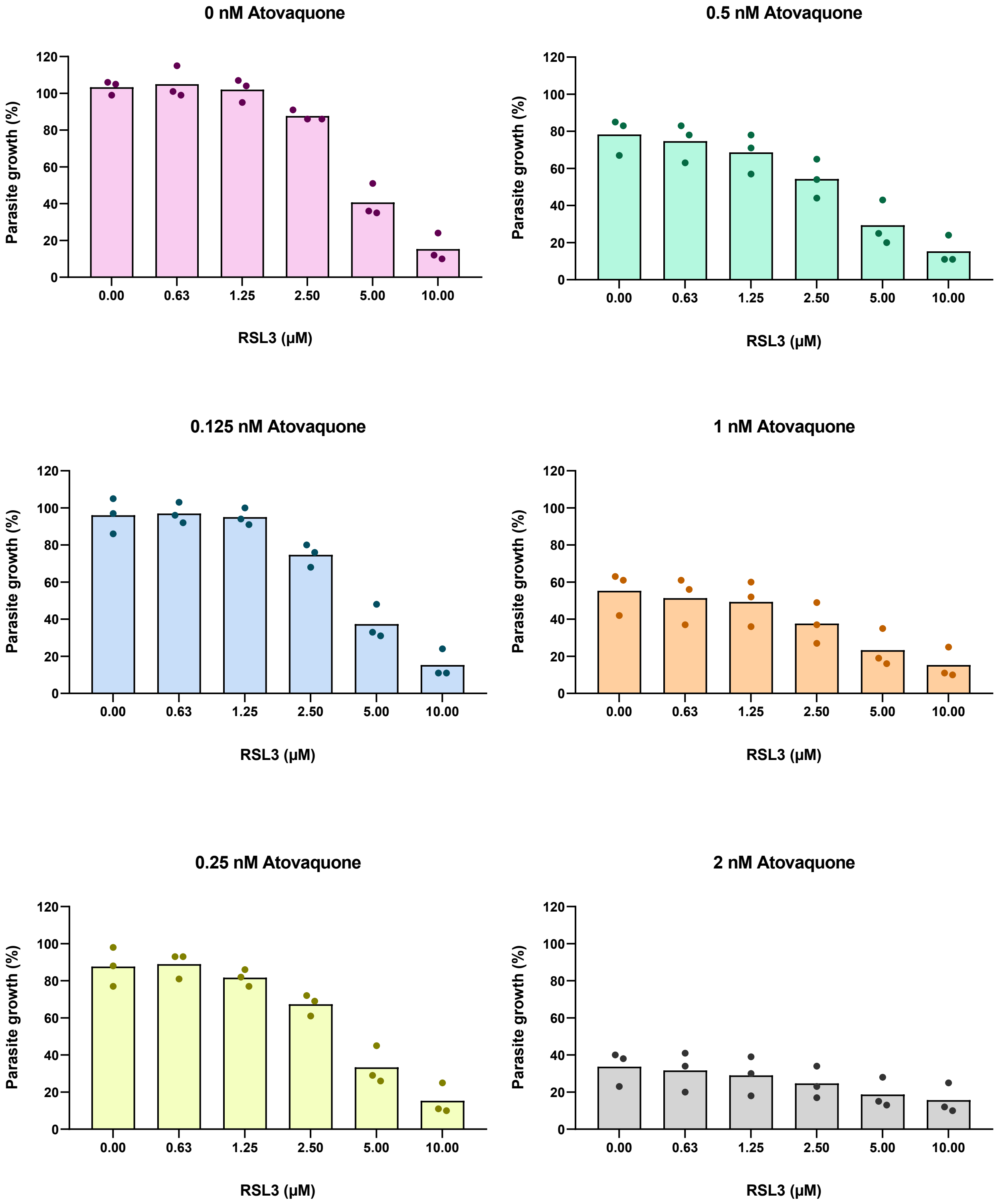

Supplemental Figure 3

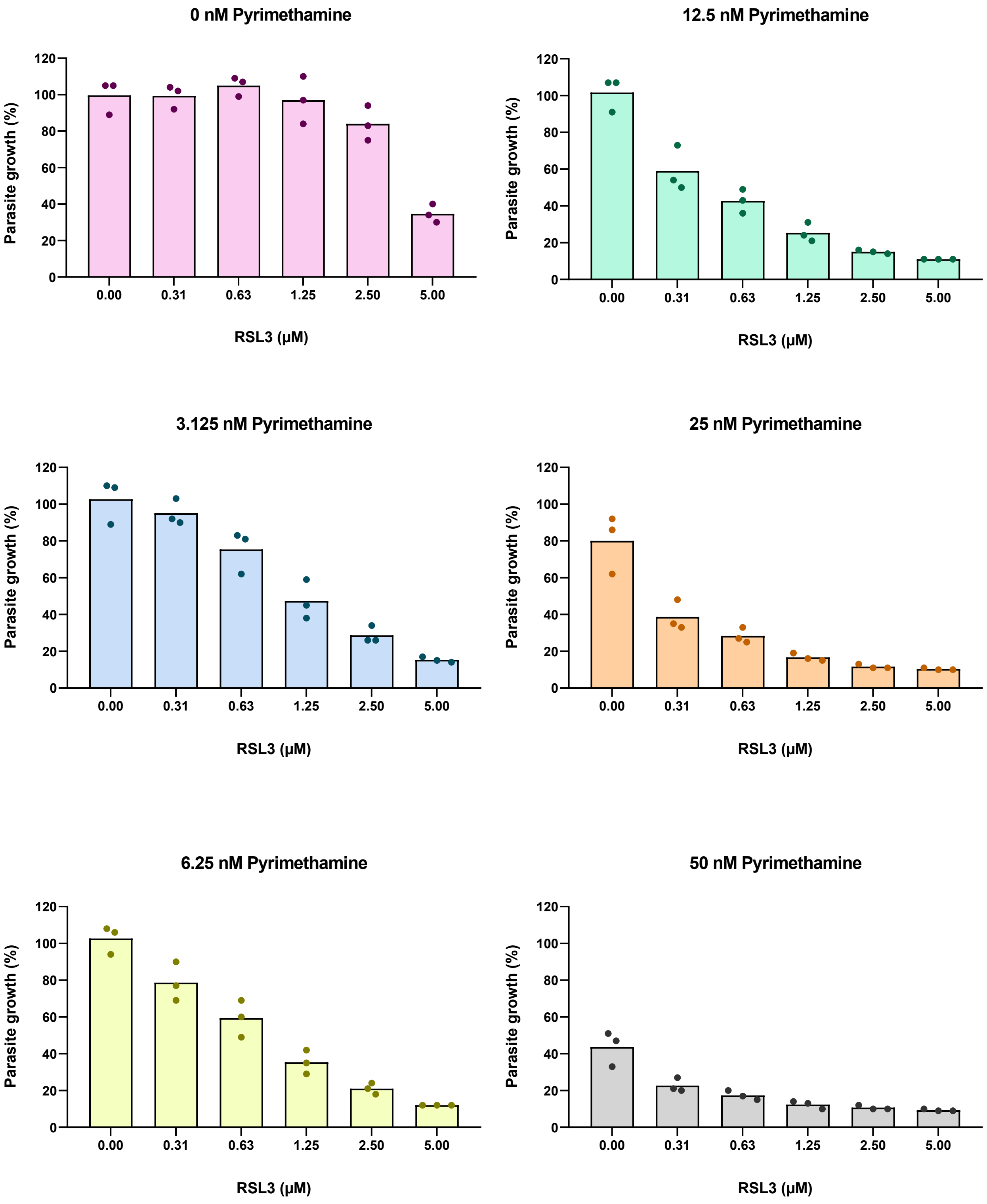

Supplemental Figure 4

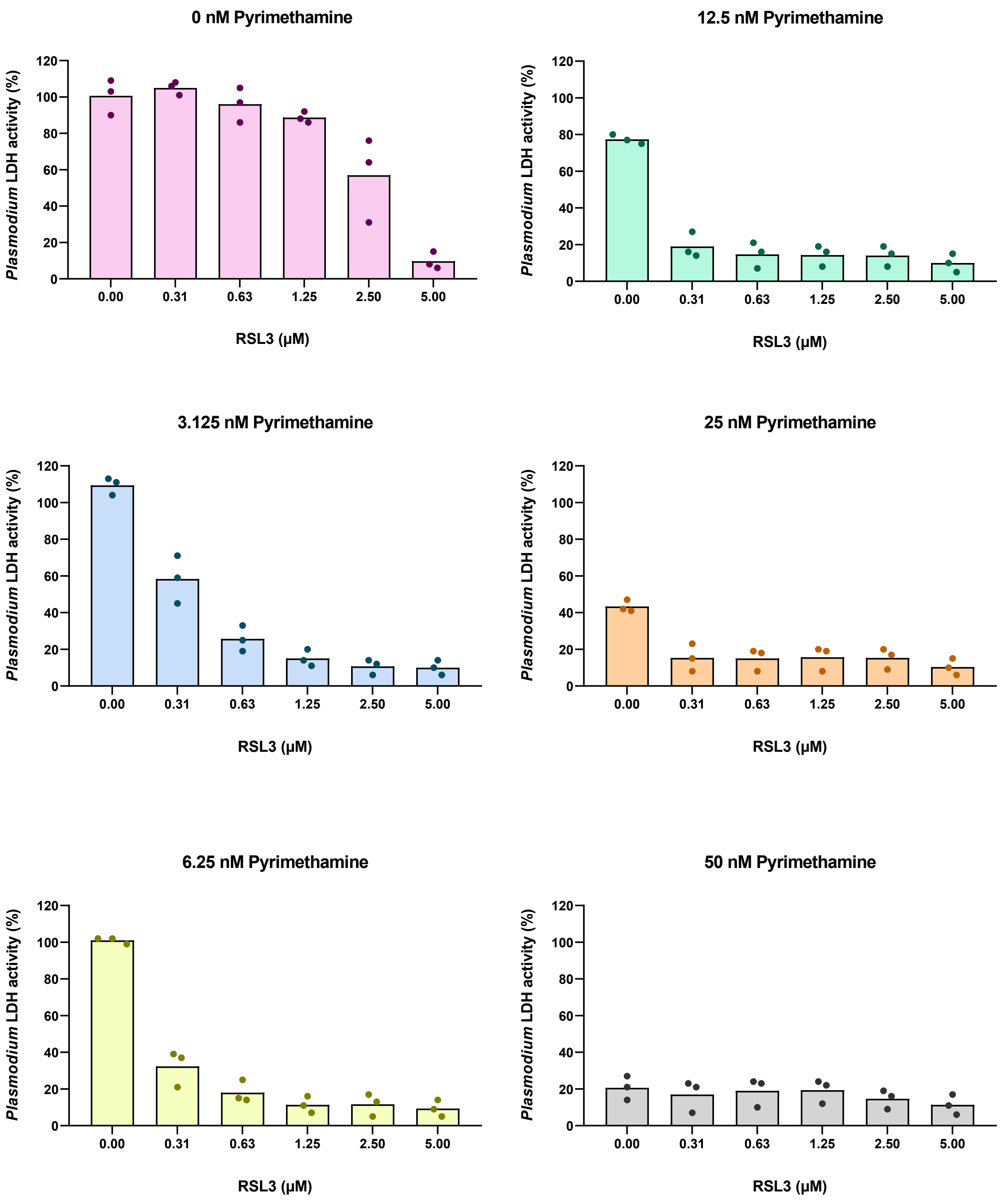

Supplemental Figure 5

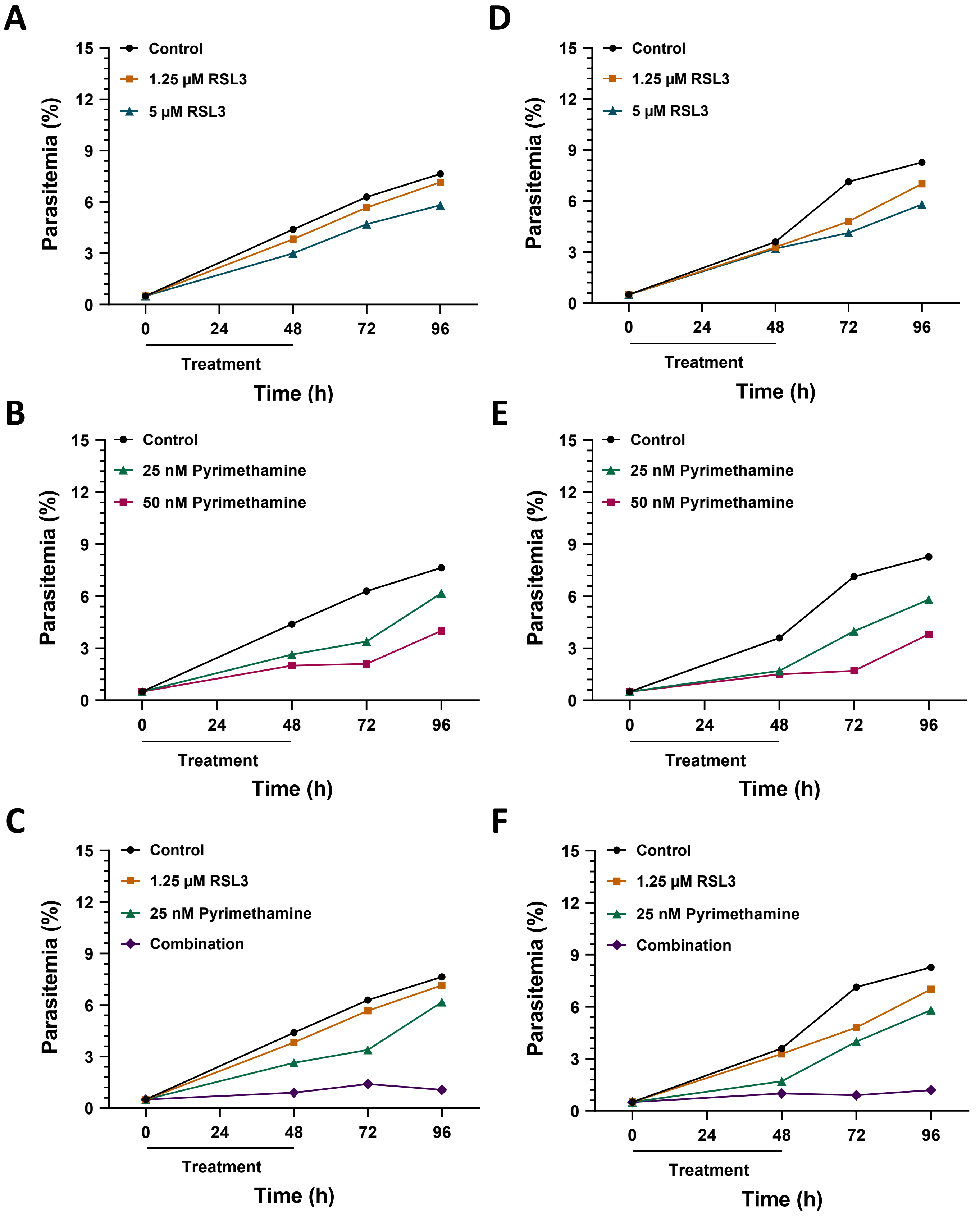

Supplemental Figure 6

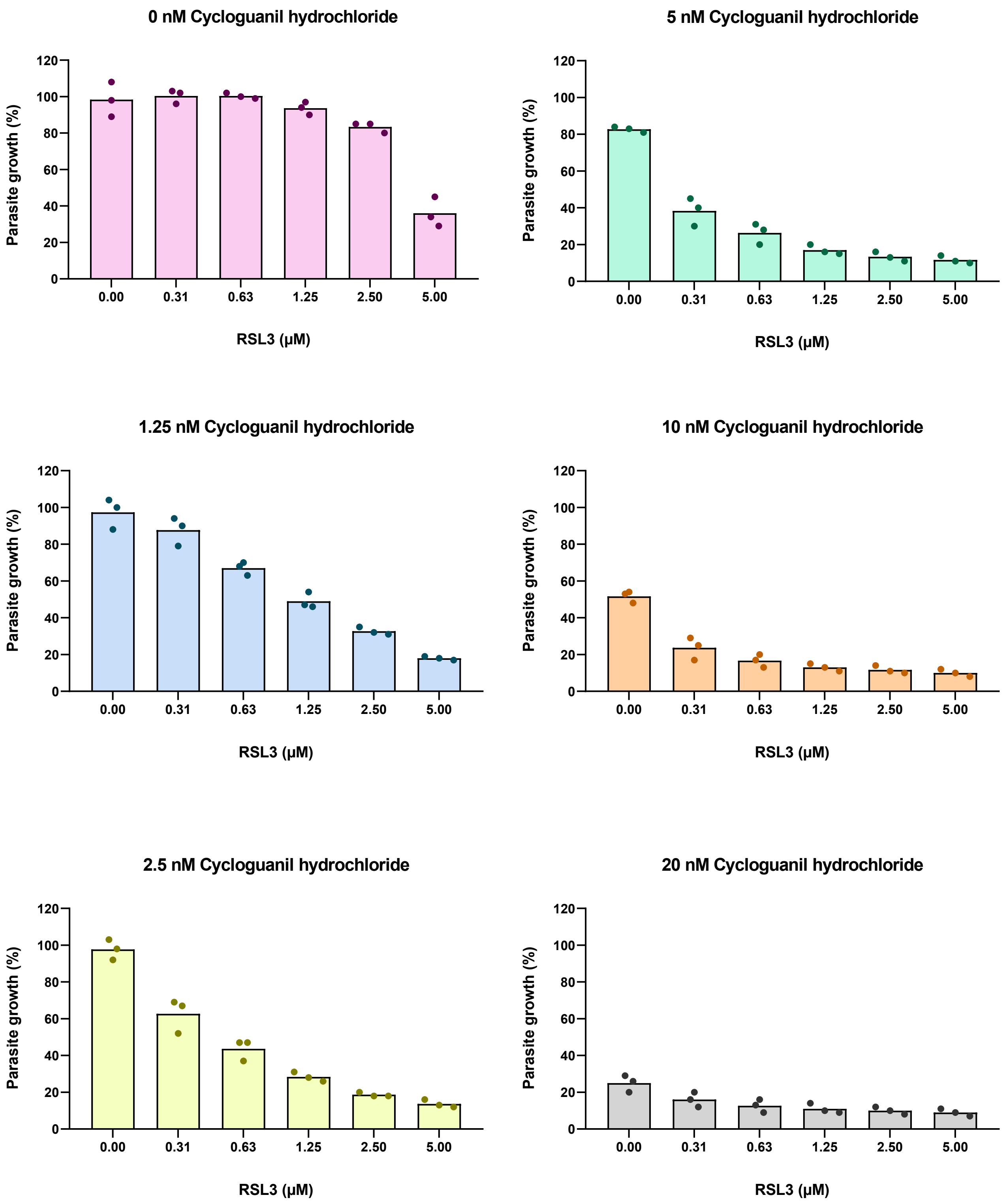

Supplemental Figure 7

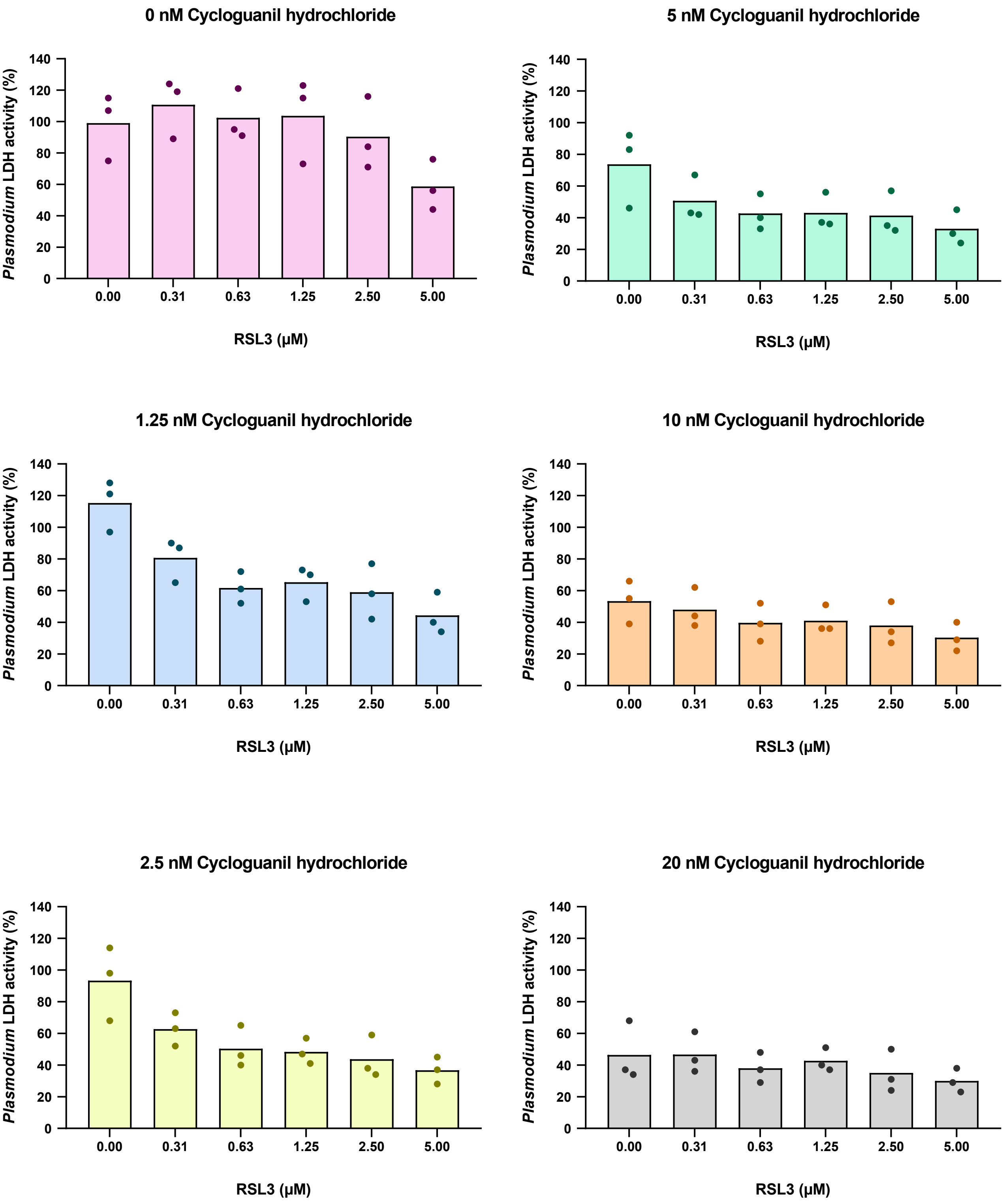

Supplemental Figure 8

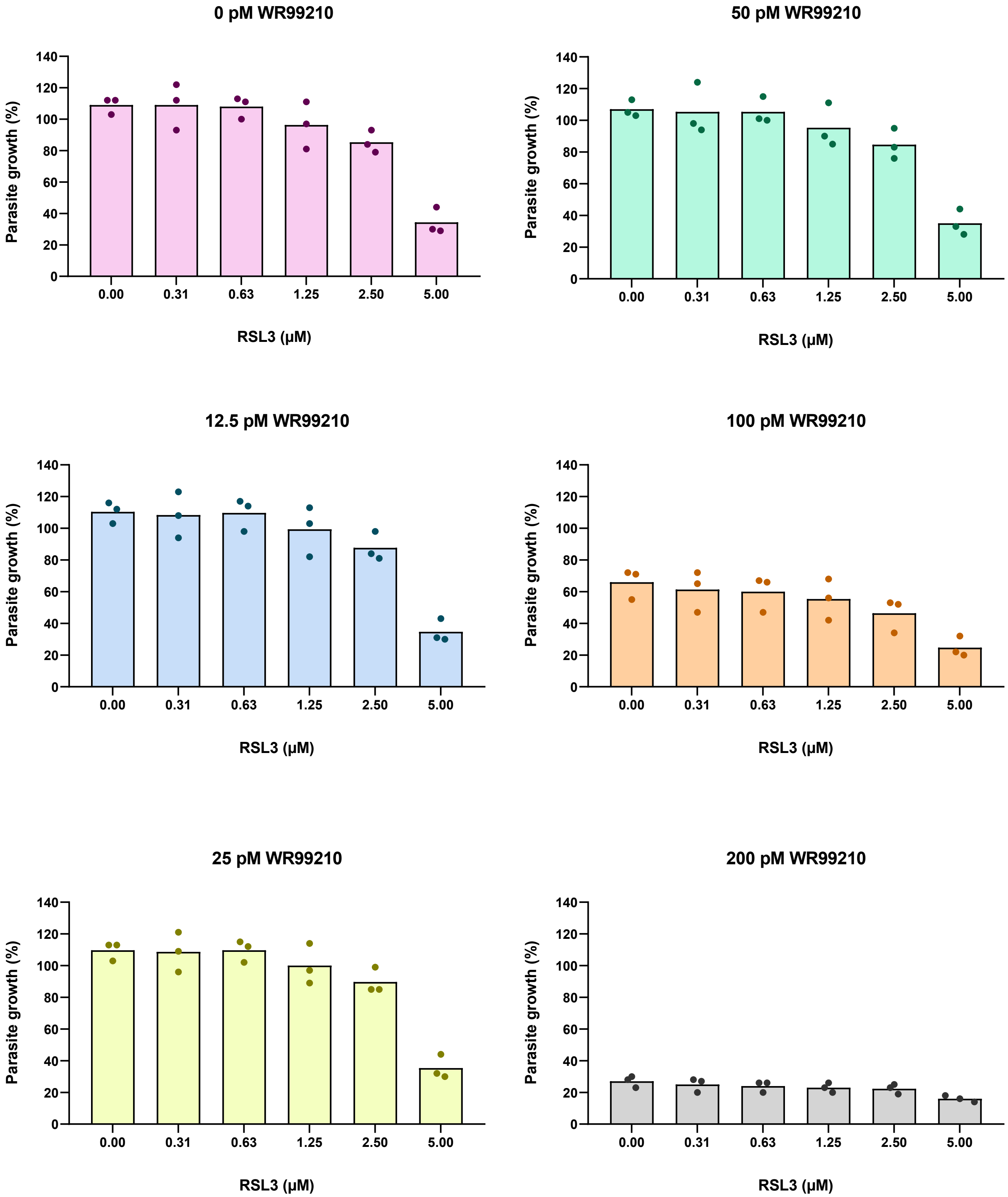

Supplemental Figure 9

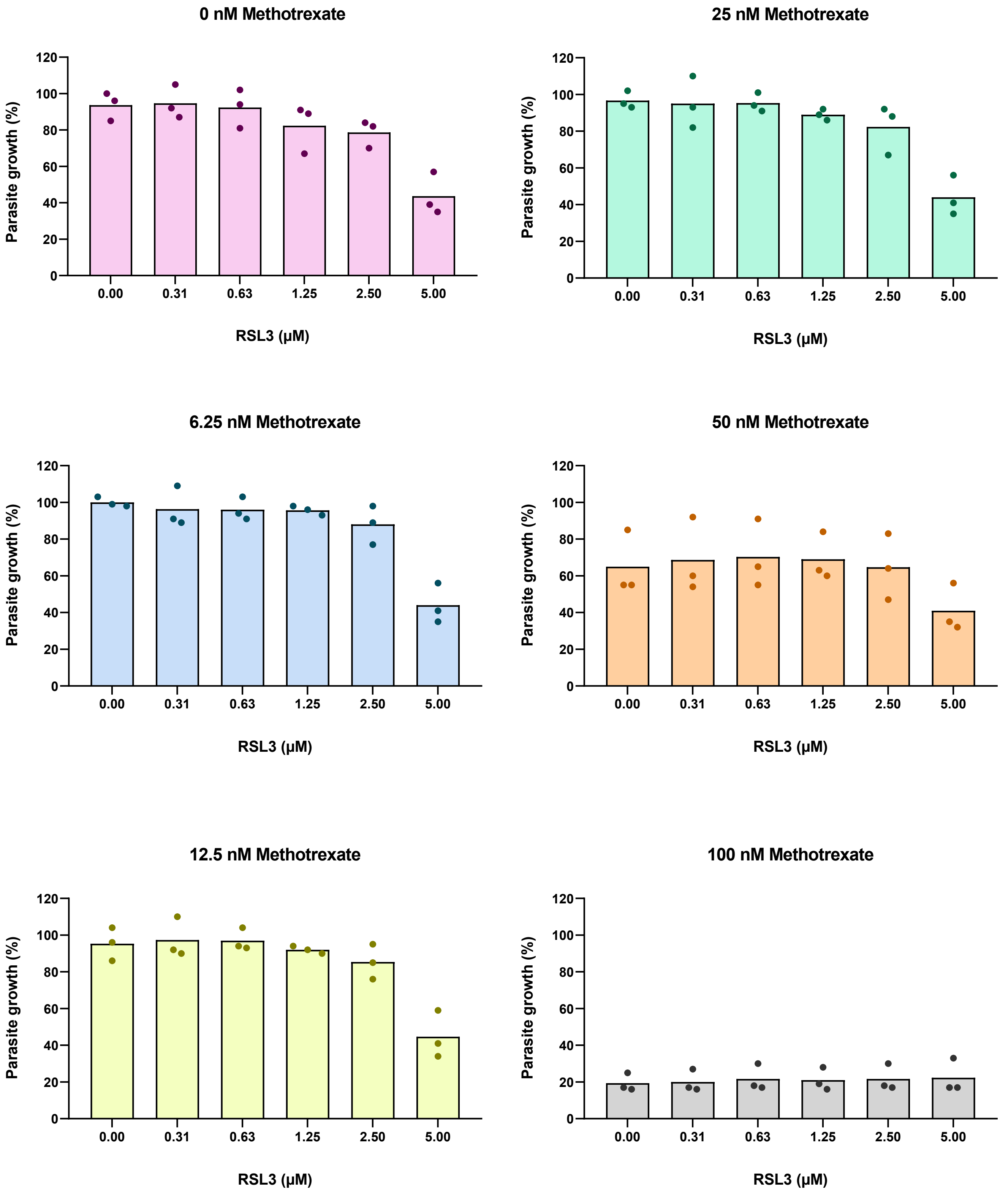

Supplemental Figure 10

A

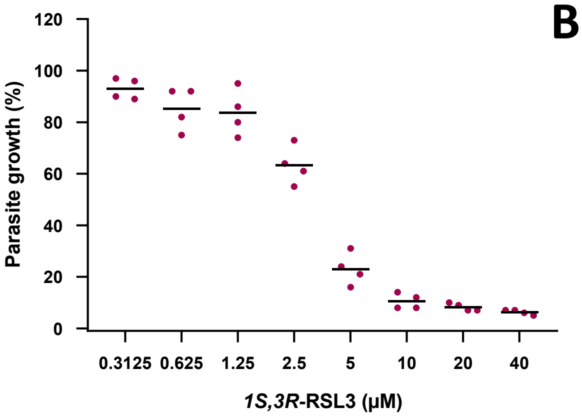

B

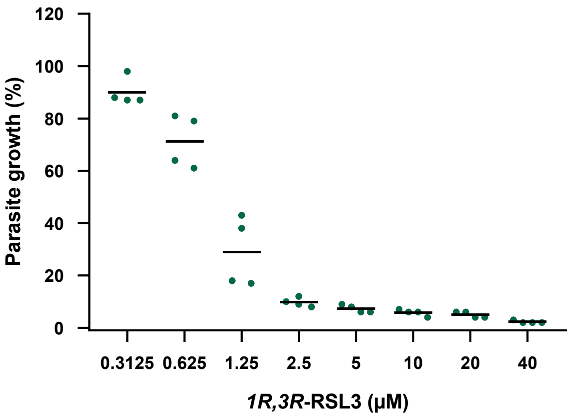

C

Compound concentration (μM)	Biological replicate	Parasite growth (%)	
		1S,3R-RSL3	*1R,3R*-RSL3
**0.3125**	1st	90	87
	2nd	96	88
	3rd	97	98
	4th	89	87
**0.625**	1st	92	61
	2nd	92	64
	3rd	75	79
	4th	82	81
**1.25**	1st	95	17
	2nd	86	18
	3rd	74	38
	4th	80	43
**2.5**	1st	61	10
	2nd	73	12
	3rd	55	8
	4th	64	9
**5**	1st	16	8
	2nd	21	9
	3rd	24	6
	4th	31	6
**10**	1st	12	6
	2nd	14	7
	3rd	8	6
	4th	8	4
**20**	1st	9	6
	2nd	10	6
	3rd	7	4
	4th	7	4
**40**	1st	7	2
	2nd	7	2
	3rd	6	3
	4th	5	2

Supplemental Figure 11

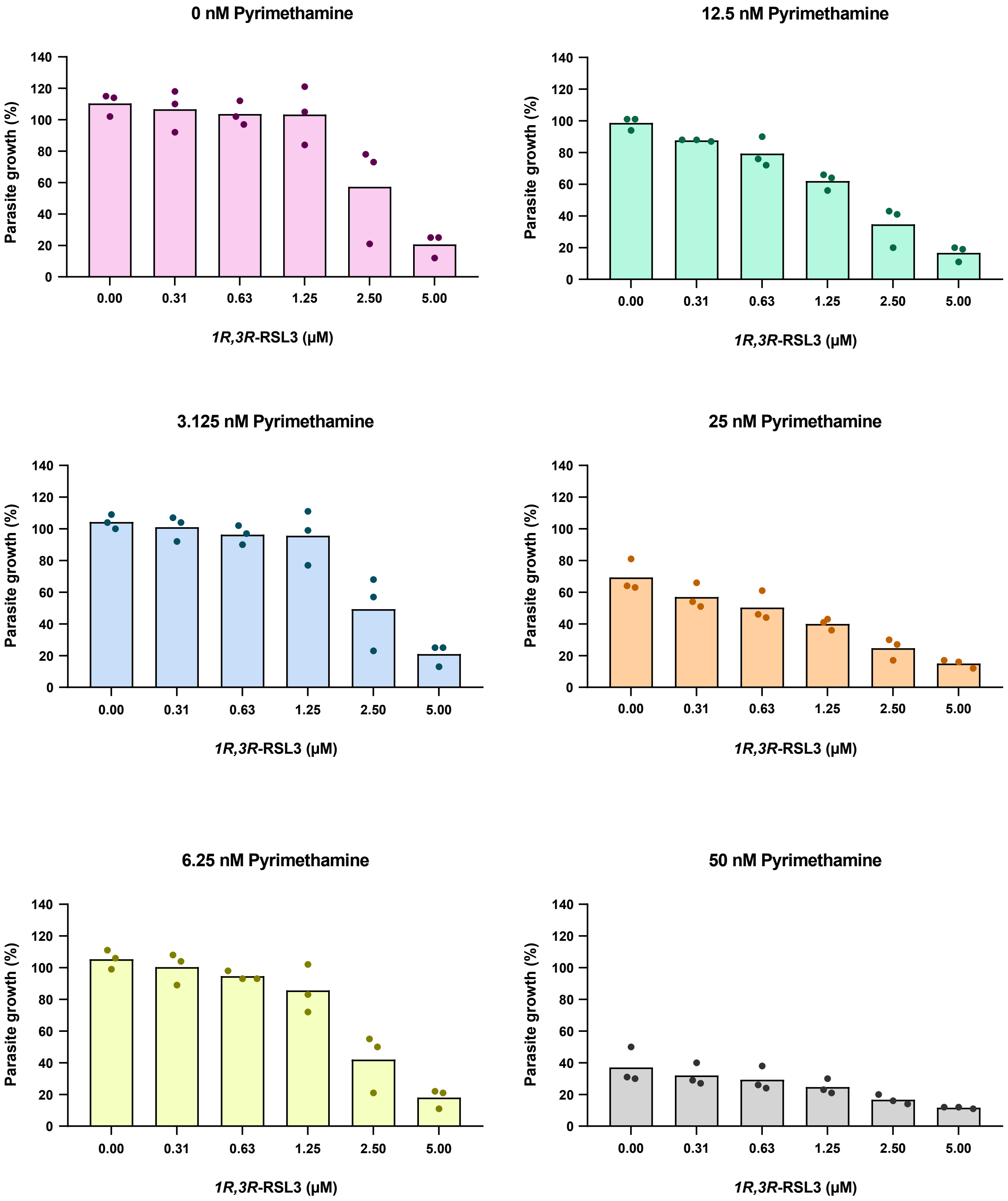

Supplemental Figure 12

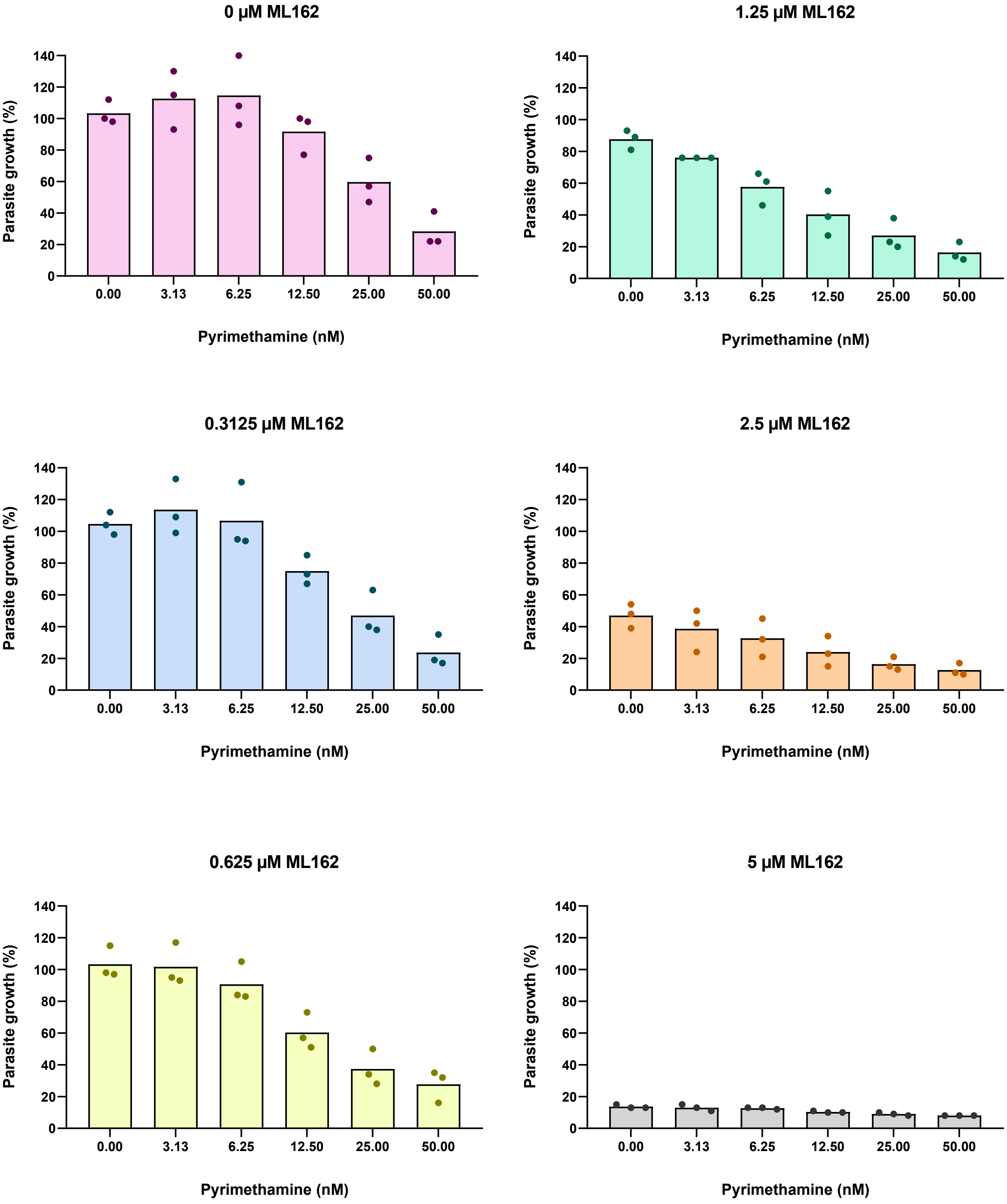

Supplemental Figure 13

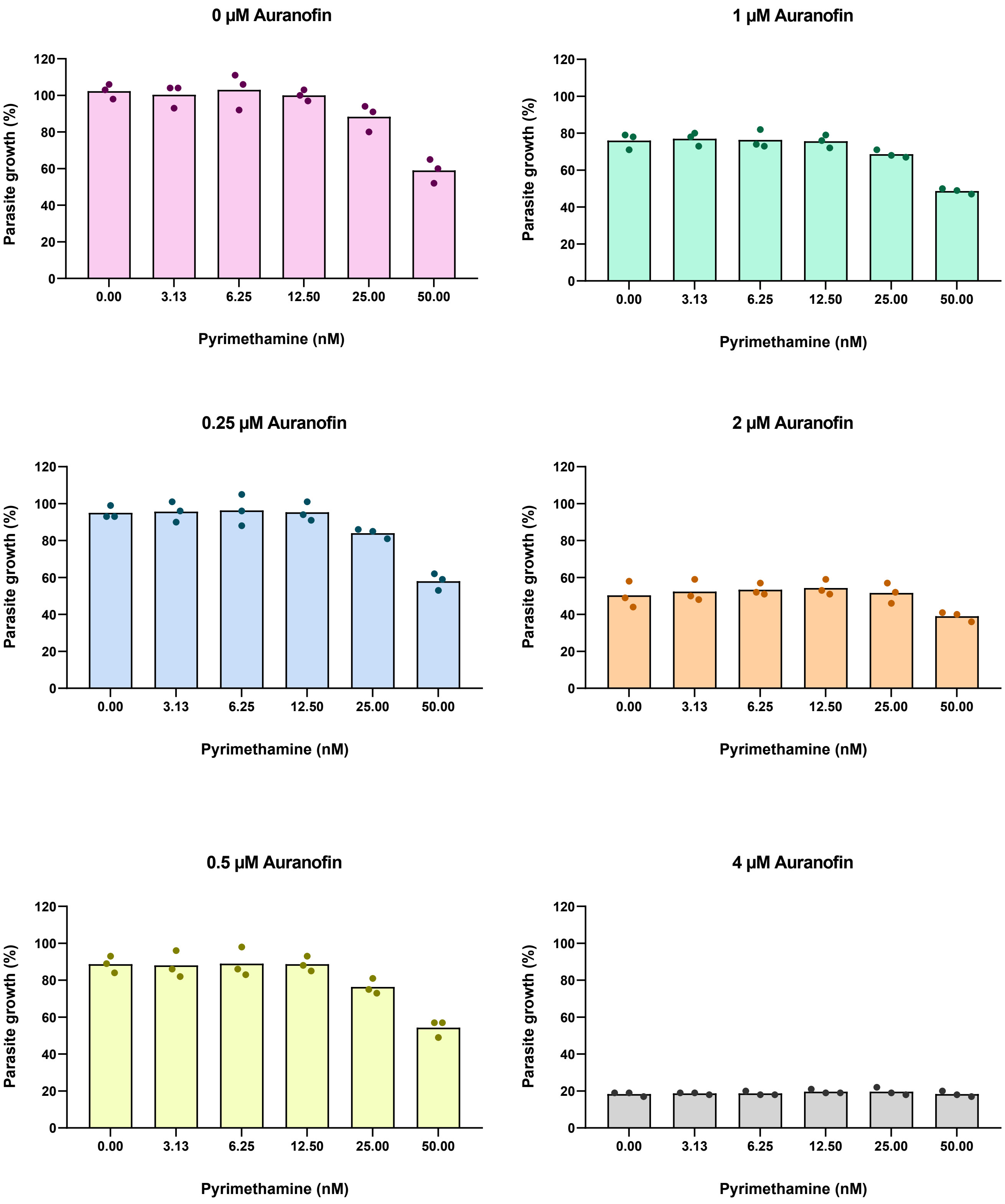

Supplemental Figure 14

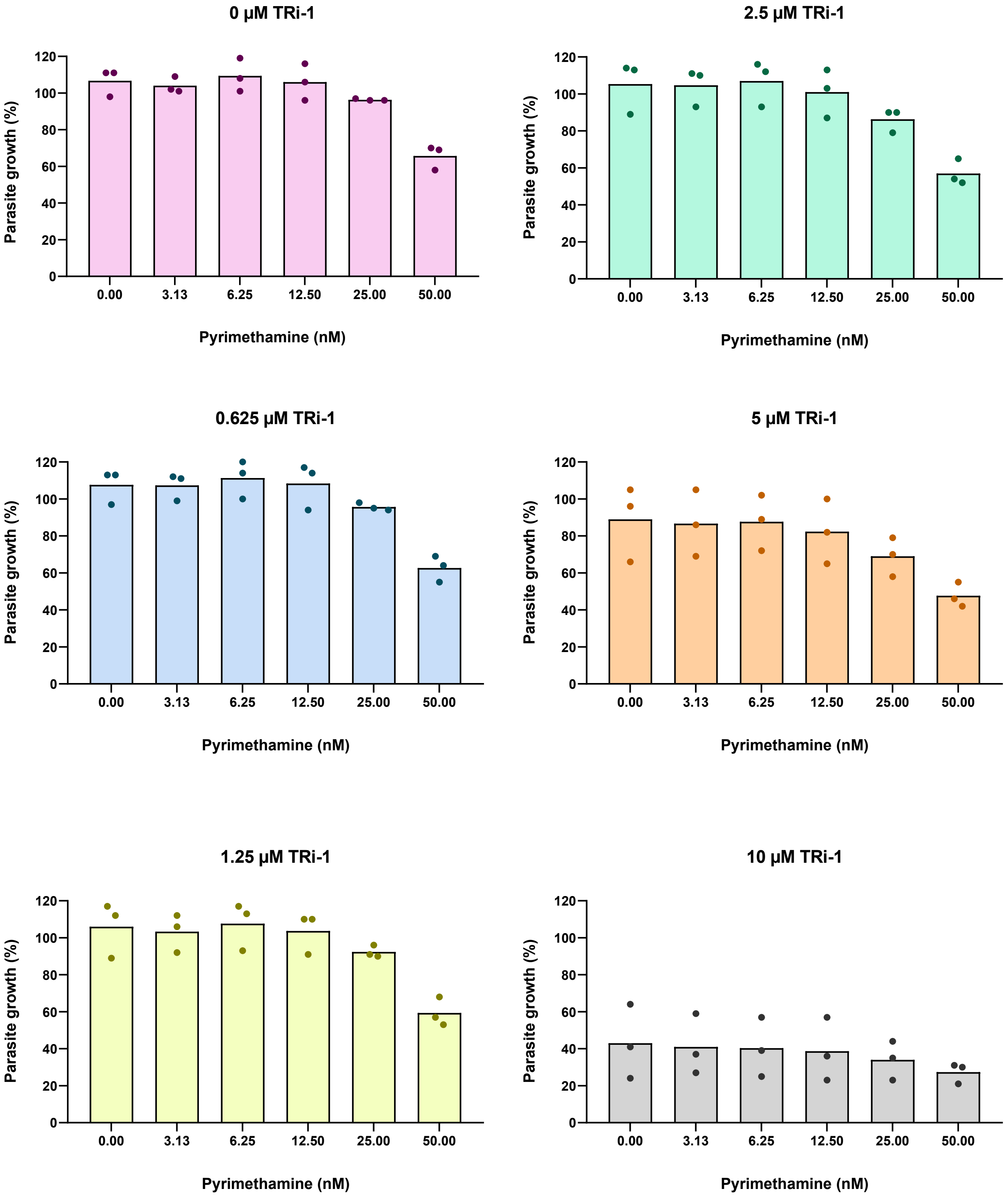

Supplemental Figure 15

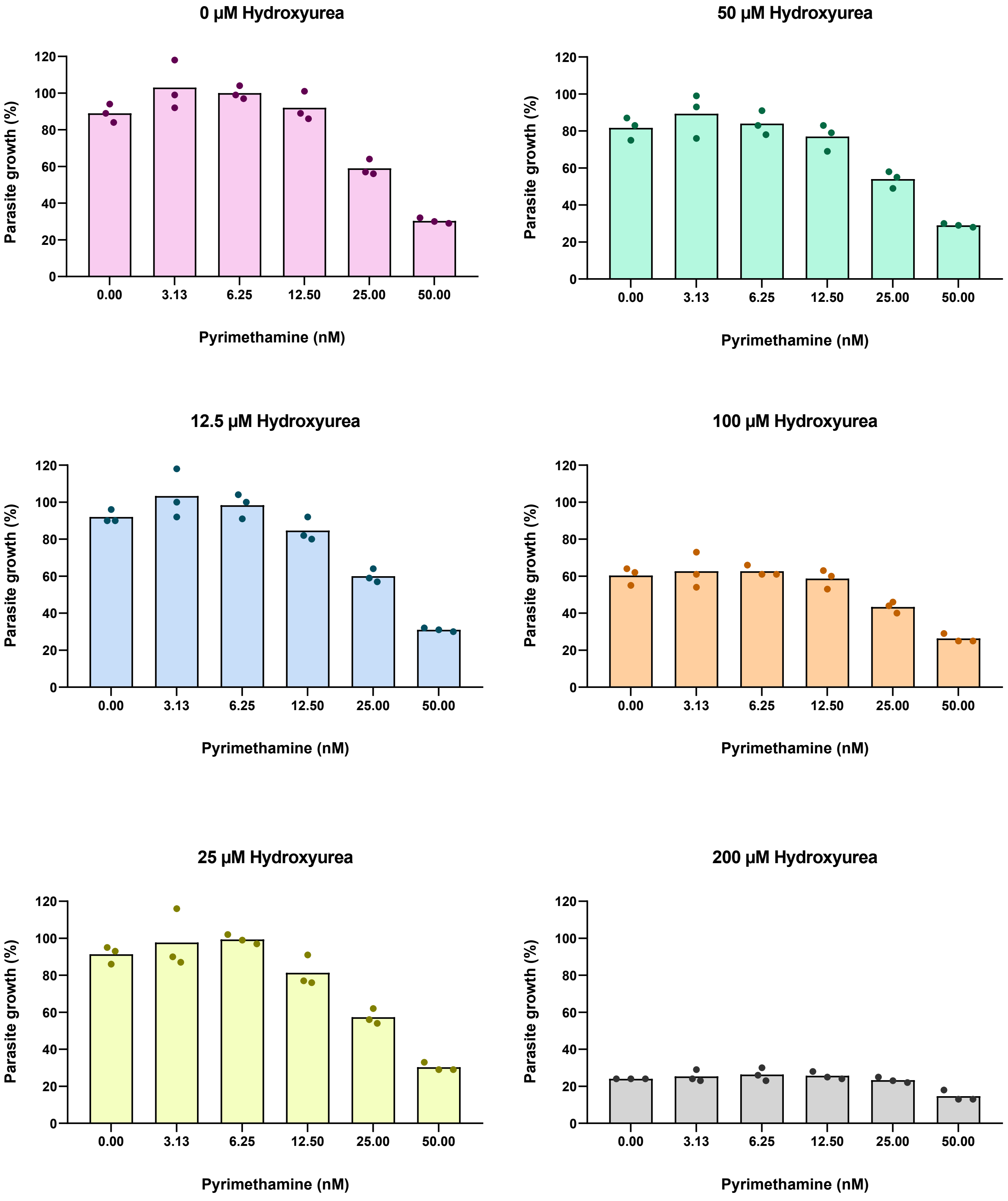

Supplemental Figure 16

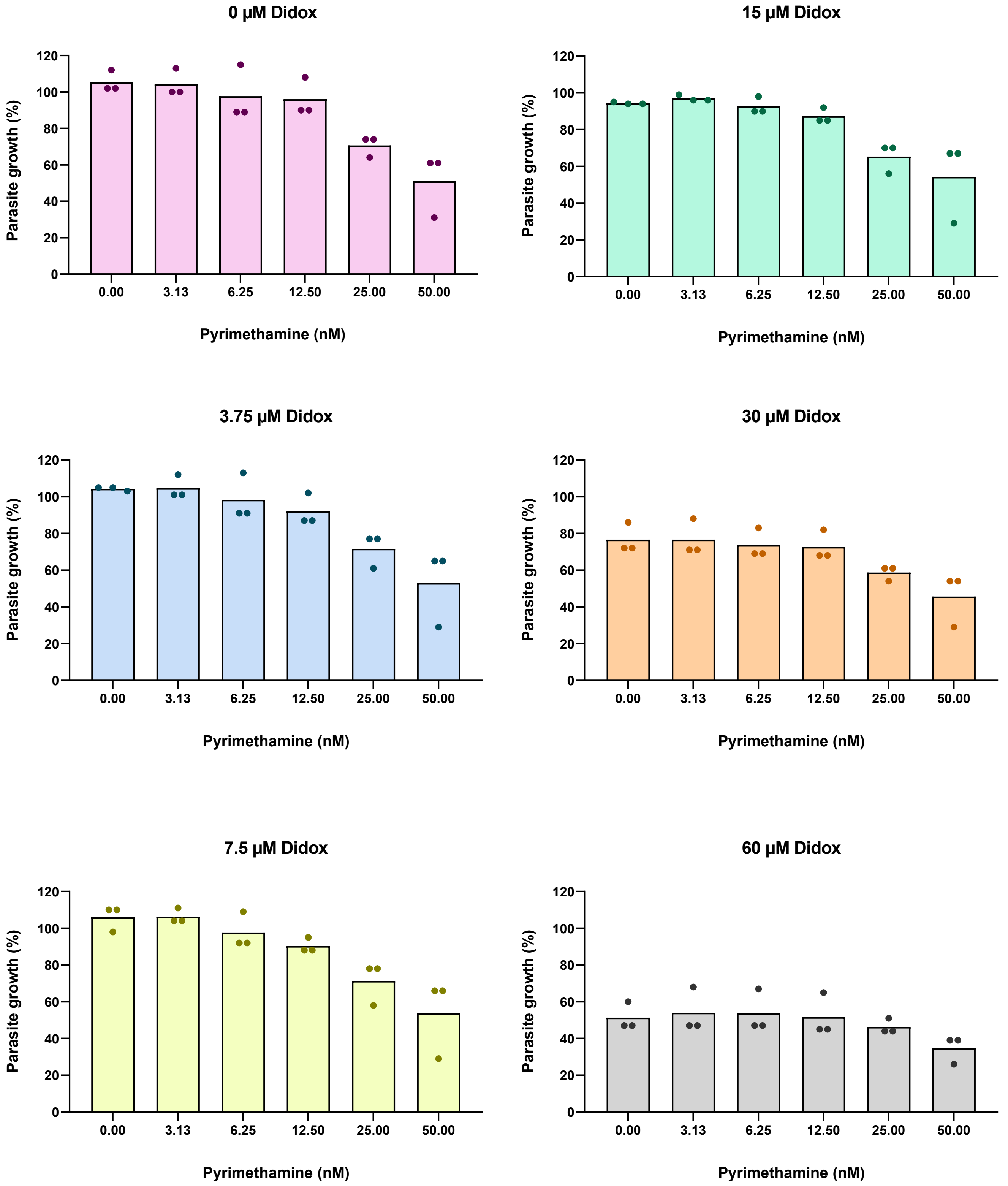

Supplemental Figure 17

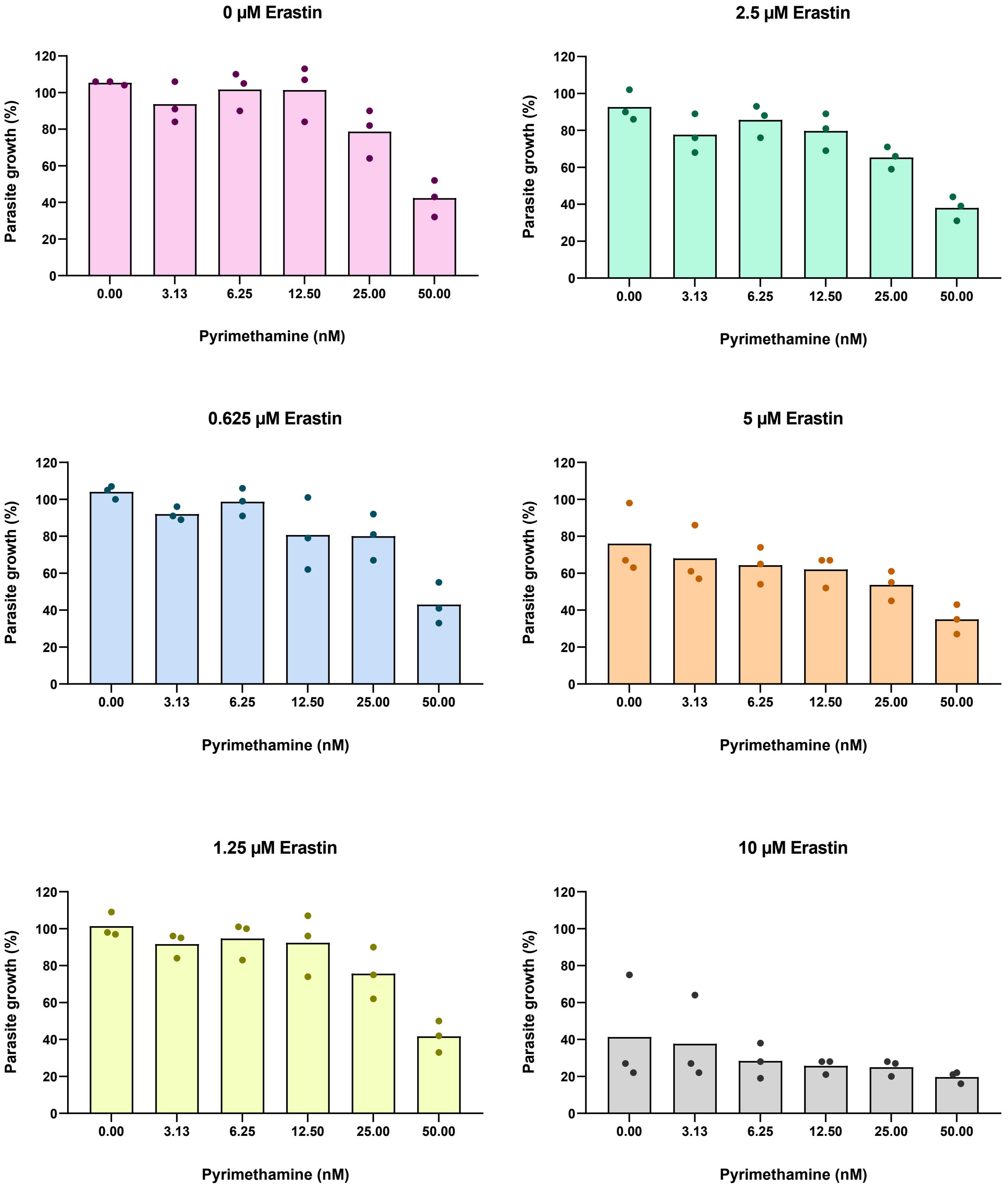

Supplemental Figure 18

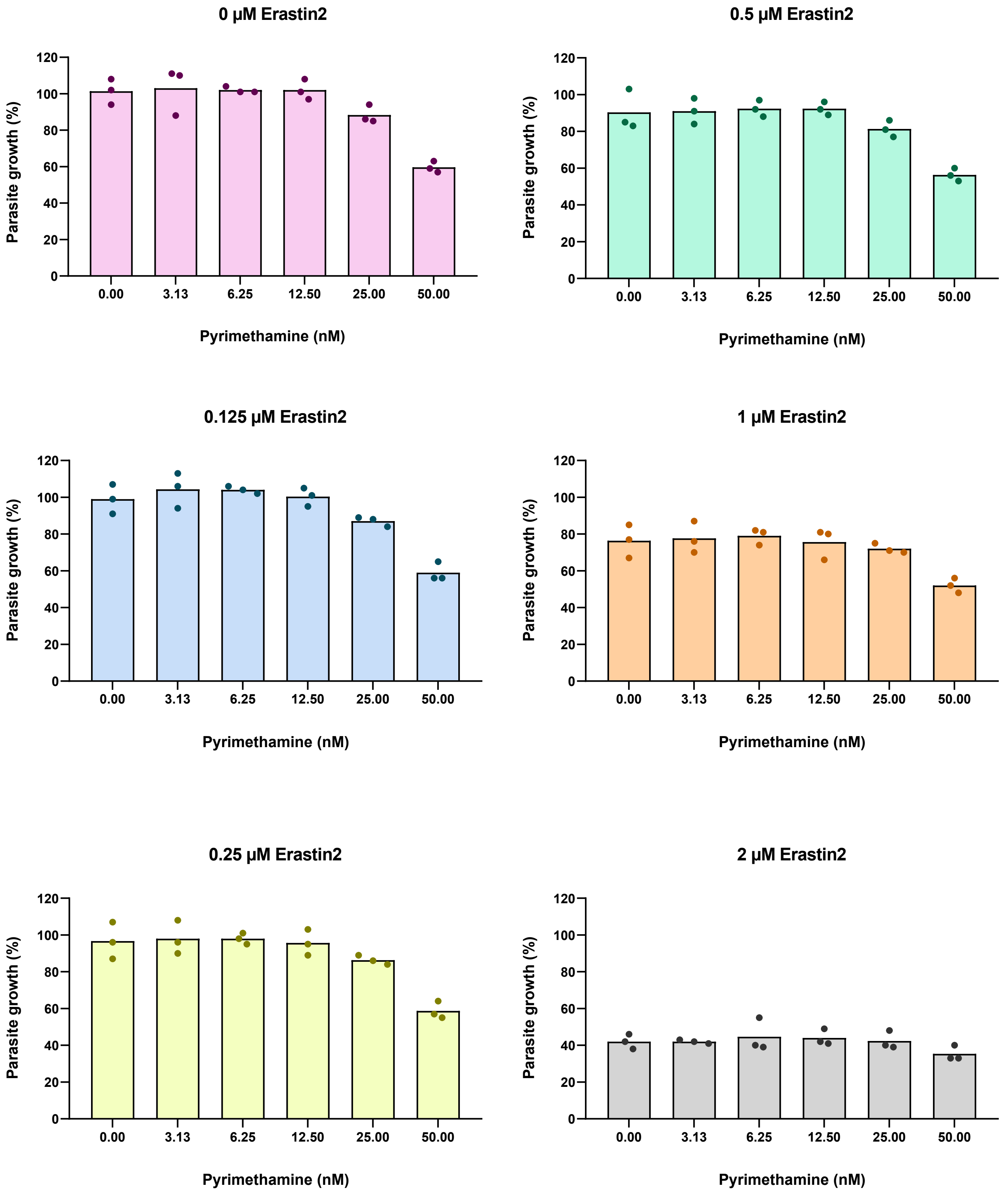

Supplemental Figure 19

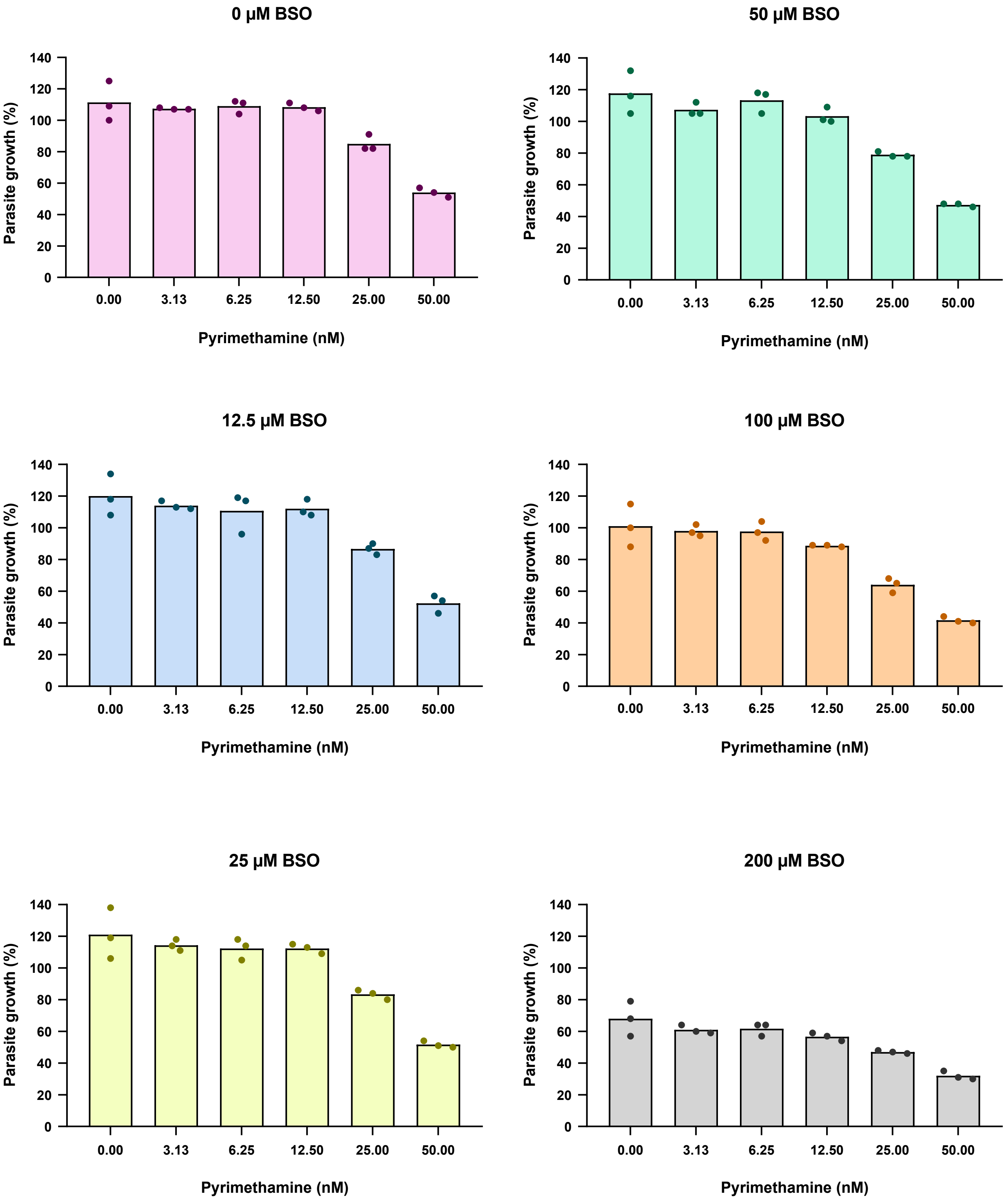

Supplemental Figure 20

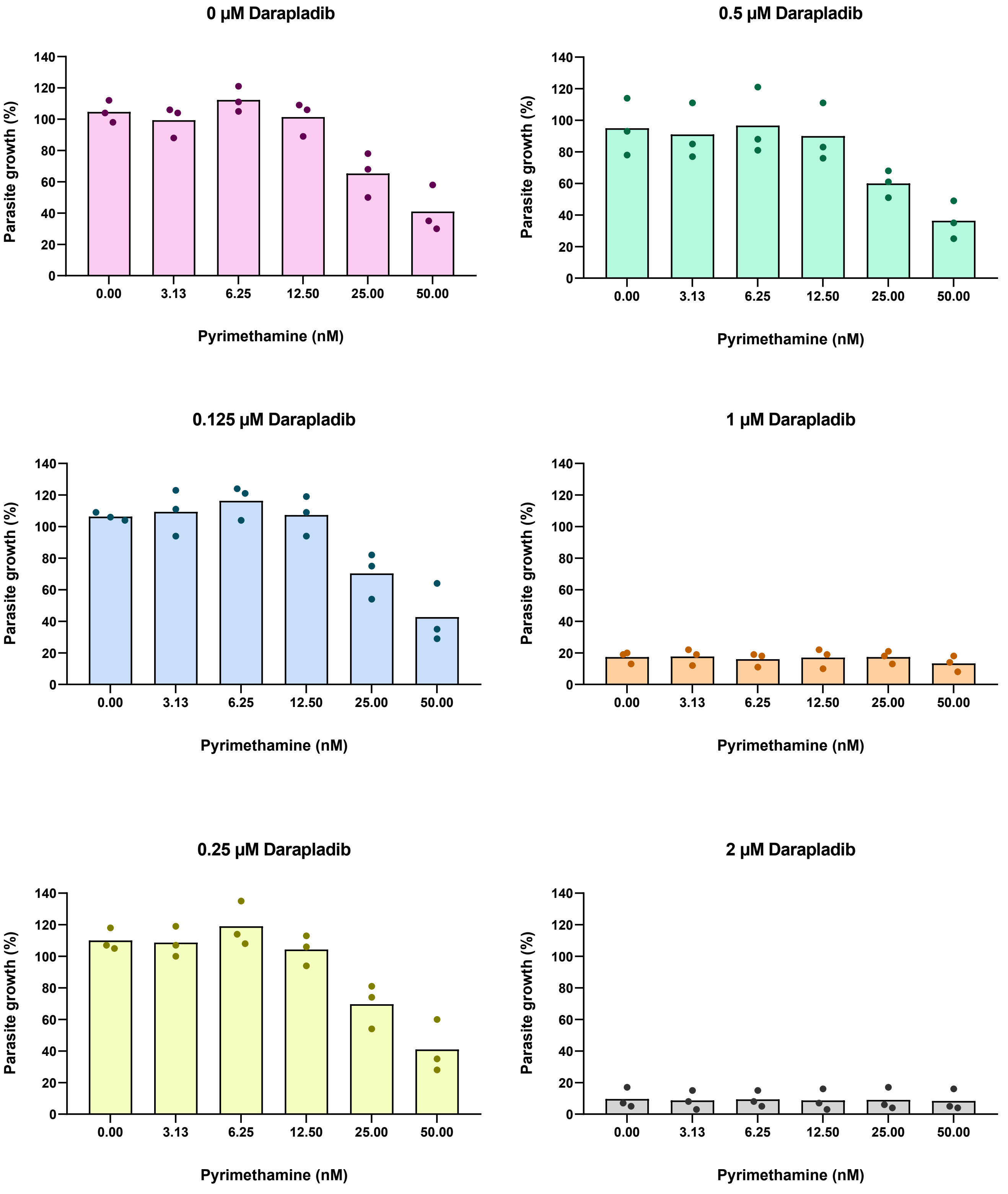

Supplemental Figure 21

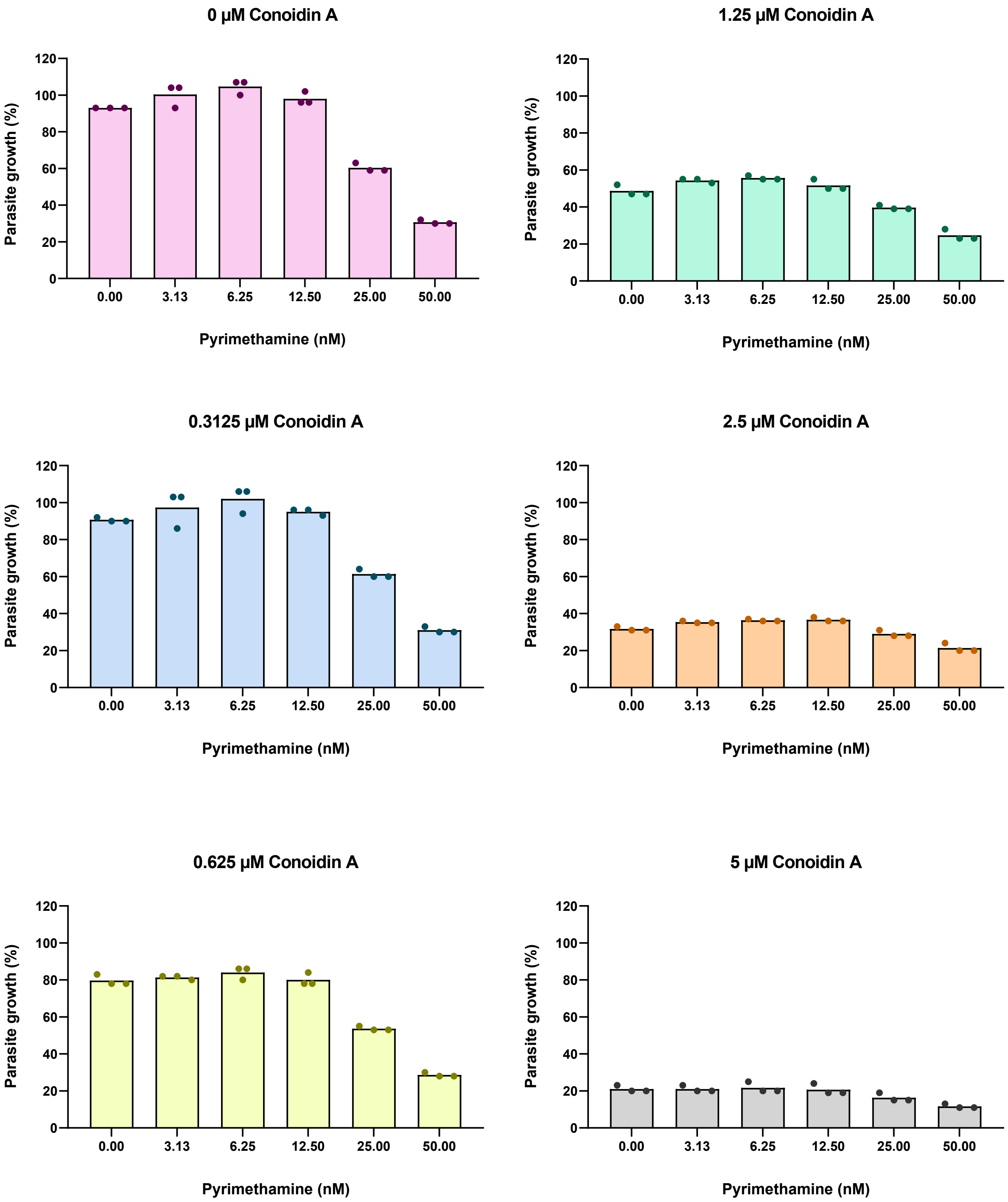

Supplemental Figure 22

A

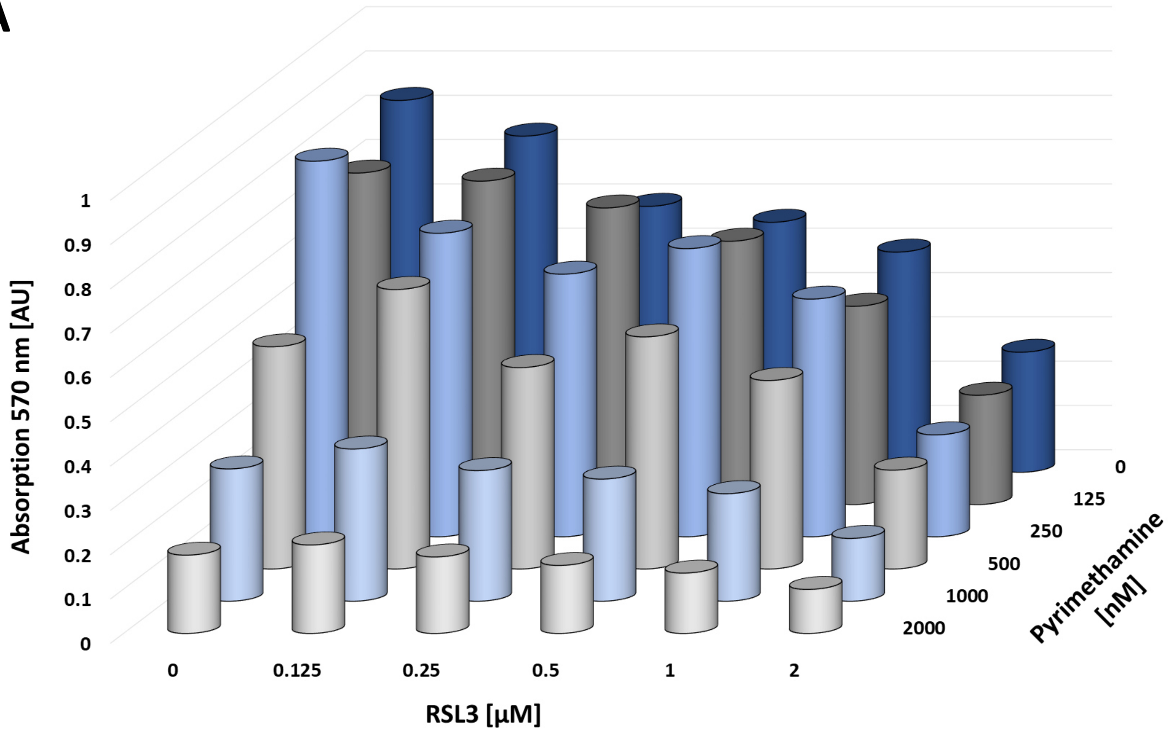

B

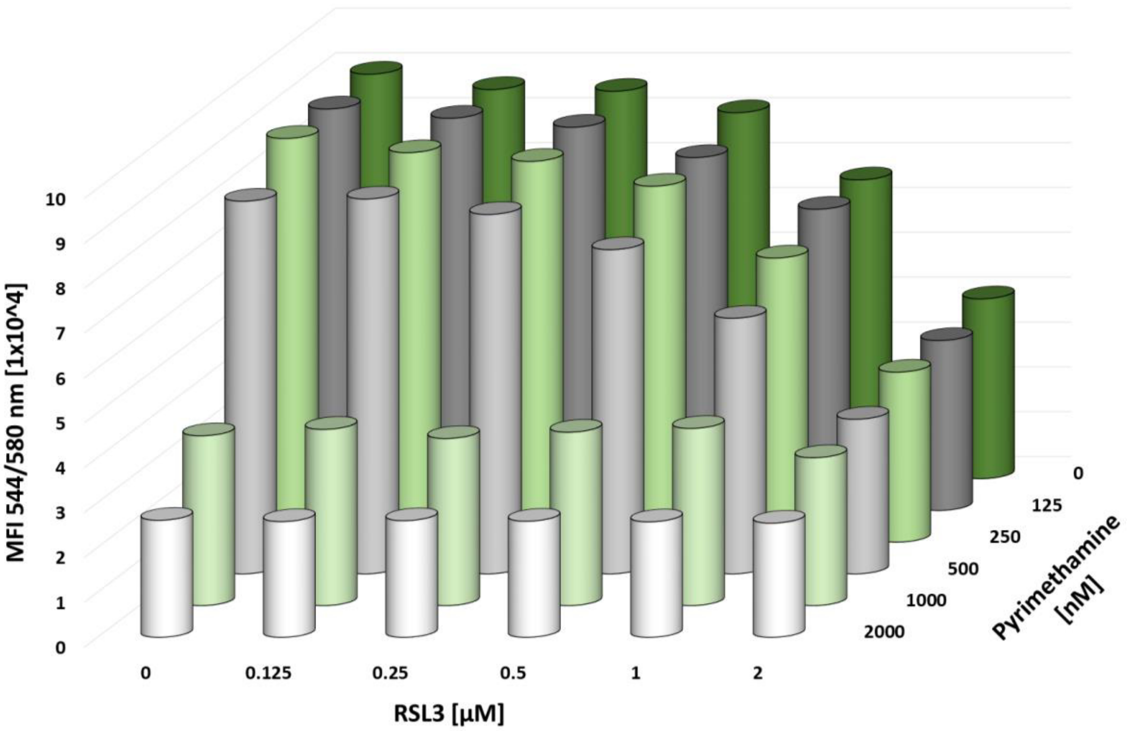

Supplemental Figure 23

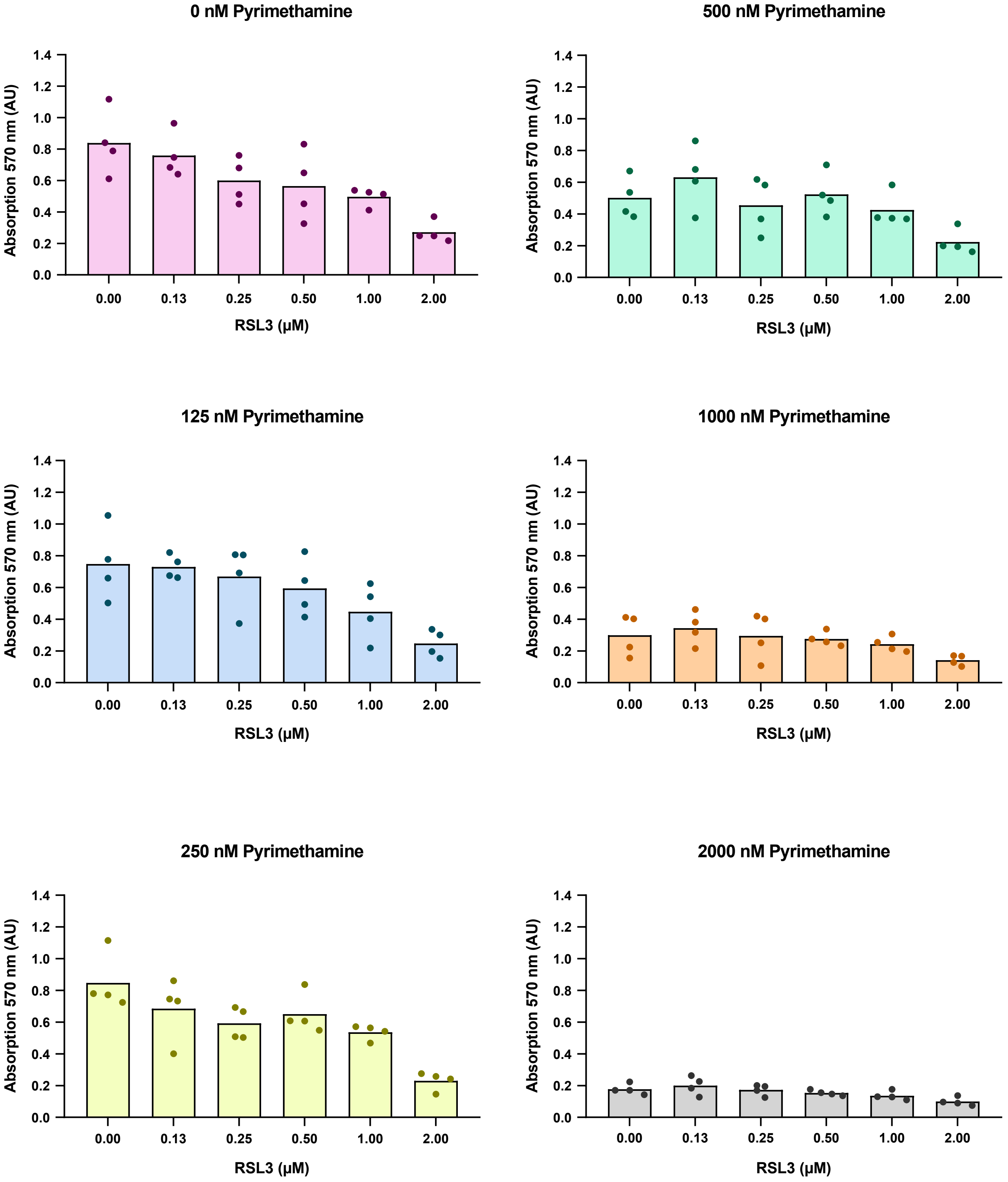

Supplemental Figure 24

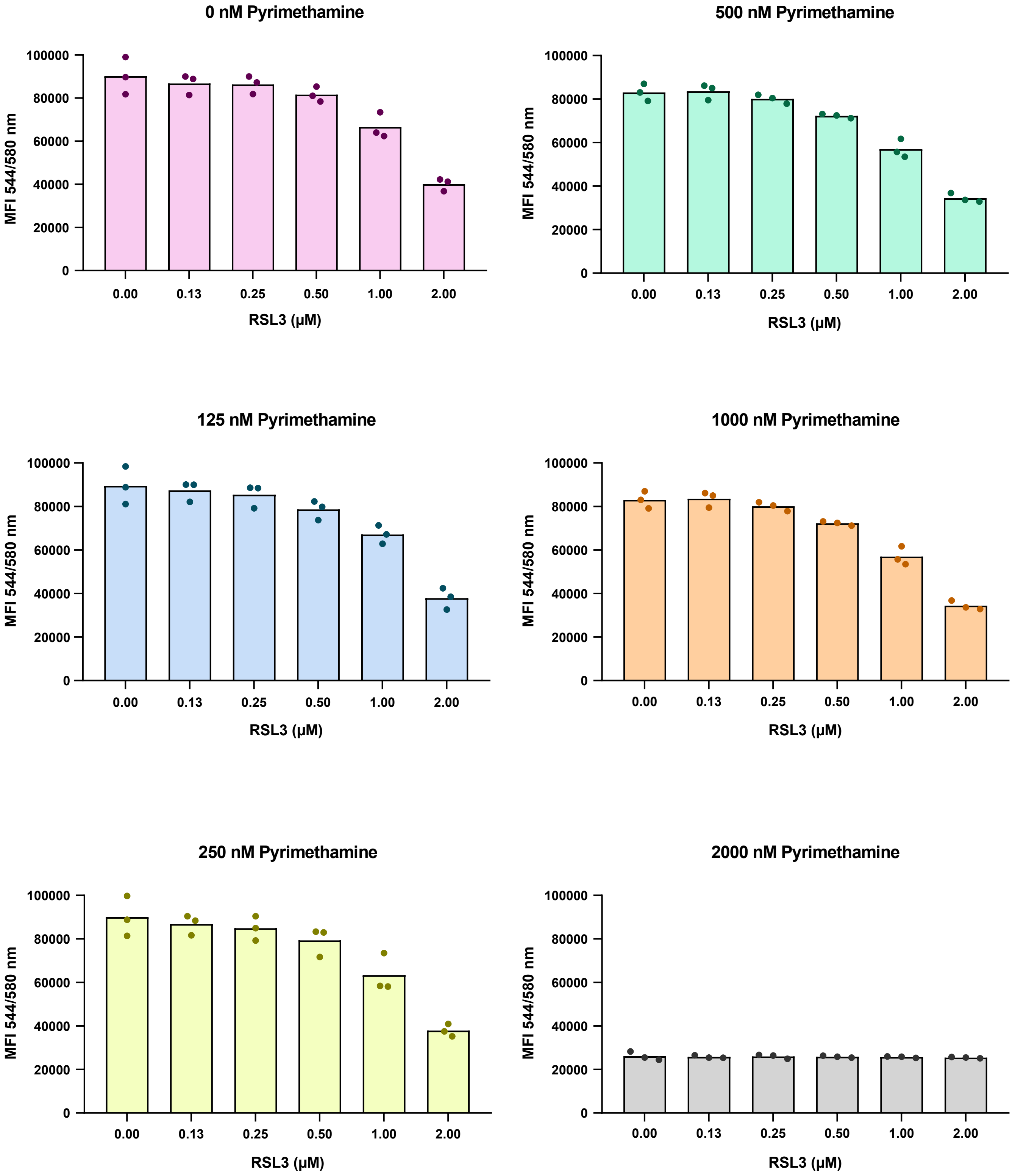

Supplement: Supplemental figures — Fig. S1 to S24. [file aac.00471-25-s0001.pdf]
